# Emergency Medical Services Provider-Perceived Alzheimer’s Disease and Related Dementias in the Prehospital Setting

**DOI:** 10.5811/westjem.18593

**Published:** 2024-11-19

**Authors:** Esmeralda Melgoza, Valeria Cardenas, Hiram Beltrán-Sánchez

**Affiliations:** * University of California Los Angeles, Jonathan and Karin Fielding School of Public Health, Los Angeles, California; † University of California Los Angeles, Latino Policy and Politics Institute, Los Angeles, California; ‡ University of California Los Angeles, California Center for Population Research, Los Angeles, California; § University of Southern California, Leonard Davis School of Gerontology, Los Angeles, California

## Abstract

**Objective:**

Our goal was to assess emergency medical services (EMS) provider-perceived Alzheimer’s disease and related dementias (ADRD) by patient sociodemographic characteristics and ZIP code tabulation areas (ZCTA) in the prehospital setting.

**Methods:**

We conducted a retrospective descriptive analysis of EMS calls with patient contact for adults ≥ 65 years of age who were provided prehospital care between February 1, 2020 and January 31, 2022, using data from the San Francisco Department of Emergency Management and the 2021 American Community Survey. Logistic regression models assessed the associated between EMS provider-perceived ADRD and patient sociodemographic characteristics, including age, race/ethnicity, incident location, and ZCTA-level socioeconomic status.

**Results:**

A total of 55,129 patient encounters were recorded, with EMS provider-perceived ADRD recorded in 4,112 (7.5%). Among cases with EMS provider-perceived ADRD, the most common primary impressions were mental disorders (17.1%), weakness (17.0%), injury (15.7%), and pain (13.1%). Increasing age was associated with higher odds of EMS provider-perceived ADRD among both sexes. Among females, EMS provider-perceived ADRD was higher among Hispanics (odds ratio [OR] 1.30, 95% confidence interval [CI] 1.11–1.52), Blacks (OR 1.20, 95% CI 1.03–1.40), Asians (OR 1.18, 95% CI 1.06–1.31), and Native Hawaiian and Pacific Islanders (OR 1.48, 95% CI 1.05–2.08]), while among males, only Asians (OR 87, 95% CI .76–.99) had lower odds, all compared to Whites. Females in low- and medium-income ZCTAs had lower odds of EMS provider-perceived ADRD relative to high-income ZCTAs, with no significant findings in males.

**Conclusion:**

Our findings suggest a higher prevalence of EMS provider-perceived Alzheimer’s disease and related dementias among minoritized and socioeconomically disadvantaged populations, including the oldest adults, and racial and ethnic minority communities. Future research and more precise data collection is needed to ensure equity for older adults who access emergency care in the prehospital setting.

Population Health Research Capsule What do we already know about this issue?

*Alzheimer’s disease and related dementias (ADRD) in older adults is projected to increase from 6.7 to 13.8 million cases between 2023 and 2060.*

What was the research question?

*How does EMS provider-perceived ADRD among older adults vary by sociodemographic characteristics and geography?*

What was the major finding of the study?

*In females, perceived ADRD was higher in minorities, including Hispanics (OR 1.30, 95% CI 1.11–1.52], P = <.05), compared to Whites.*

How does this improve population health?

*Revealing sociodemographic and geographic variations among subpopulations of older adults advances our understanding of EMS provider-perceived ADRD in the prehospital setting,.*

## INTRODUCTION

Alzheimer’s disease and related dementia (ADRD) cases among adults ≥65 years of age in the United States are projected to increase from 6.7 million to 13.8 million between 2023 and 2060.^1^ The projected increase in ADRD cases is partially attributed to the increasing population of adults ≥65 years of age, which is expected to rise from 58 million to 88 million between 2021 and 2050.^1^ Although older age does not directly cause ADRD, it is a significant risk factor for disease development.^2^ The estimated ADRD prevalence is 5% for ages 65 to 74, 13.1% for ages 75 to 84, and 33.3% for ages ≥85, indicating a heightened disease burden with increasing age.^1^

Existing literature provides evidence of the presence of health disparities in ADRD incidence and prevalence based on sex, race, ethnicity, and socioeconomic status.^3^ Alzheimer’s disease and related dementias are more prevalent in older adult women than men.^1^ ^,^ ^3^ While women, on average, have longer life expectancies at age 65 than men, this finding does not fully account for their increased ADRD risk.^1^ ^,^ ^3^ Among older adults, Black and Hispanics are 2 and 1.5 times more likely, respectively, to have ADRD compared to Whites.^3^ Dementia incidence, including ADRD, is highest among Blacks and American Indian or Alaska Natives (AIAN), intermediate for Hispanics, Native Hawaiian and other Pacific Islanders (NHOPI), and Whites, and lowest among Asian-Americans.^4^ Lower education is associated with an increased risk of ADRD, which suggests socioeconomic disparities.^5^

### Importance

The existing literature suggests that there are health disparities in ADRD detection, treatment, and research.^3^ ^,^ ^6^ ^,^ ^7^ ^–^ ^9^ Hispanic and Black older adults report worse cognitive function and more functional limitations at ADRD diagnosis compared to Whites, suggesting detection at a later stage of the disease.^6^ Racial and ethnic disparities in ADRD-targeted treatment are mixed.^6^ ^,^ ^7^ Non-adherence and discontinuation rates of ADRD medications are higher among Hispanic and Black older adults compared to Whites, partially due to challenges with access and cost of healthcare.^6^ ^,^ ^7^ The generalizability of ADRD research is also limited due to the under-representation of certain racial and ethnic groups, including Hispanic older adults in clinical trials.^8^ ^,^ ^9^ Given the robust literature suggesting racial and ethnic disparities in ADRD detection, treatment, and research, assessing healthcare services provided to persons with ADRD in the US emergency care system is critical.

Previous studies suggest that the US emergency care system serves as an entry point for older adults with ADRD.^10^ ^,^ ^11^ Most research, however, focuses on in-hospital emergency care provided in the emergency department (ED).^11^ Older adults with ADRD have higher rates of ED visits, 30-day ED revisits, and inpatient admissions compared to older adults without ADRD.^11^ In the ED, 40% to 64% of visits by older adults with ADRD result in an inpatient hospital admission, averaging a stay of 6.5 days.^11^ Among older adults with ADRD, the most common reasons for ED visits include accidents and behavioral disturbances.^12^ Older adults with ADRD who are female, ≥85 years of age, and who have multiple medical comorbidities are more likely to use the ED.^12^

### Goals of This Investigation

While studies suggest that older adults with ADRD are more likely to use in-hospital emergency services, including the ED, few studies have examined the provision of emergency medical services (EMS) to older adults with suspected or confirmed ADRD.^10^ ^–^ ^12^ One study that assessed the provision of EMS to older adults with ADRD reported more ambulance transports to an ED for this population, compared to older adults without ADRD.^13^ In this study, we aimed to 1) assess the most common primary and secondary EMS-provider impressions listed in the prehospital setting for persons with EMS provider-perceived ADRD; and 2) analyze EMS provider-perceived ADRD by patient sociodemographic characteristics and ZIP code tabulation areas (ZCTA).

## METHODS

### Study Setting

We used 9-1-1 EMS data from the consolidated city-county of San Francisco, CA. San Francisco is one of 58 counties in California with a population of 815,201 as of 2021.^14^ San Francisco has 46.9 square miles of land, making it the smallest county in the state in terms of square mileage but the most densely populated with 18,629 people per square mile.^14^ San Francisco is one of the most racially and ethnically diverse counties in the state, with a racial composition of 49.9% White, 39.8% Asian, 15.7% Hispanic, 6.7% Black, 1.7%, AIAN, 0.9% NHOPI, and 14.2% two or more races.^14^ Adults ≥65 years of age and older comprise 17.5% of the county’s population, an estimate that is projected to increase over the next three decades.^14^ ^,^ ^15^

### Study Design

In this retrospective descriptive analysis we used data from the San Francisco Department of Emergency Management and the 2021 American Community Survey five-year estimates. The San Francisco Department of Emergency Management dataset contains patient sociodemographic characteristics (eg, age, race, ethnicity, sex), incident ZIP code, provider primary and secondary impressions, and EMS provider-perceived ADRD for all patient encounters. The 2021 American Community Survey five-year estimates include ZCTA-level estimates and median household income.^16^ This study is exempt from institutional review board review.

The EMS data from the San Francisco Department of Emergency Management includes all patient encounters submitted by the San Francisco Fire Department, King American Ambulance Company, and American Medical Response, Inc. These 9-1-1 agencies include both municipal and private entities. The EMS dataset includes all patient encounters with adults ≥65 years of age who had an incident in San Francisco between February 1, 2020–January 31, 2022. The study timeframe began on February 1, 2020, because this is the date when the California data repository began to reliably populate electronic patient care report data after a change in their analytic service.

A patient encounter is defined as a 9-1-1 call where there is an encounter between a patient and an EMS provider. A single person may have multiple patient encounters during the study period. Although all patient encounters involve an EMS response, not all EMS responses result in a patient encounter. The EMS responses without a patient encounter are excluded from this study, as their associated electronic patient care reports lack details on key variables, including patient sociodemographic characteristics, provider impressions, and EMS provider-perceived ADRD. Examples of EMS responses without a patient encounter include canceled calls or instances where patients were not found at the scene. This study also excludes interfacility transports, concentrating instead on EMS provider-perceived ADRD among non-institutionalized older adults in the community.

The American Community Survey is an ongoing national survey conducted by the US Census Bureau on a random sample of the population.^16^ The survey is administered annually, with over 3.5 million households contacted every year to participate.^16^ Selected households complete the survey via mail, telephone, or in-person interviews, providing data on a range of social, economic, demographic, and housing topics across several geographics.^16^ For smaller geographics, including ZCTAs, five-year estimates are published to increase statistical data reliability and confidentiality. In this study we used median household income across ZCTAs from the 2021 American Community Survey five-year estimates.

### Measurements

The dependent variable is presence (coded 1) or absence (coded 0) of EMS provider-perceived ADRD. This outcome is a combination of ADRD diagnoses disclosures by patients and caregivers, and perceived ADRD by the EMS provider. It is impossible, however, to disentangle confirmed with perceived ADRD from the data in the electronic patient care reports, although this is currently the best source of data available in the prehospital setting. Five independent variables including age, sex, race/ethnicity, ZCTA-level median household income, and incident ZIP code, are included based on previous literature.^17^ ^–^ ^19^ Patient sociodemographic characteristics and incident ZIP code are recorded by EMS providers in the electronic patient care reports. The method used to collect sociodemographic information for each patient encounter is not available in the dataset, although it is likely that this data is obtained through a combination of patient self-report and provider report.

Age is coded using the following categories: 65–69 (reference group), 70–74, 75–79, 80–84, 85–89, 90–94, and 95+. Gender is coded 1 for female and 0 for male. The race and ethnicity variable is coded 1 to 6 with the following categories: White (reference group); Black; Asian; NHOPI; AIAN; and Hispanic. Median household income is coded into three groups: $0–$104,299; $104,400–$146,999; and ≥$147,000 (reference). Among the 27 ZIP codes in San Francisco, seven (94104, 94105, 94108, 94111, 94129, 94130, and 94158) had less than 40 EMS provider-perceived ADRD cases. These were pooled for reliable coefficient estimates in the statistical models. ZIP codes with over 40 incidents were sequentially coded from 1 to 20: 94102, 94103, 94107, 94109, 94110, 94112, 94114 to 94118, 94121 to 94124, 94127, and 94131 to 94134. Primary and secondary provider impressions in the electronic patient care reports are based on the International Classification of Diseases, 10^th^ Revision, codes and are available to EMS providers a priori.

### Outcomes

The first objective assesses how EMS provider-perceived ADRD is recorded in the prehospital setting, with a focus on the most common primary and secondary provider impressions. The second objective analyzes EMS provider-perceived ADRD by patient sociodemographic characteristics and ZCTAs.

### Data Analysis

We calculated descriptive statistics of the most common primary and secondary provider impressions for patient encounters with EMS provider-perceived ADRD. Bivariate analyses were performed to assess the associations between the independent variables and the outcome variable (presence or absence of EMS provider-perceived ADRD). We used chi-square tests for categorical variables, while continuous variables were analyzed using Student *t*-tests. Stepwise logistic regression was used to identify the independent associations between the five independent variables (age, sex, race/ethnicity, incident ZIP code, and median household income) and the outcome variable. (See Web Appendices Table 1.) These regression models show statistically significant differences by sex in the odds of EMS provider-perceived ADRD, and we thus proceeded to estimate stratified models by sex. (See Web Appendices Tables 2 and 3.) For this study, we calculated 95% confidence intervals (CI), and a *P*-value of <.05 was used to represent statistical significance. We used STATA v 17.0 (StataCorp, College Station, TX) for statistical analyses.^20^

In conducting this retrospective descriptive analysis, we adhered to several best practices for retrospective chart review as suggested by Worster and Bledsoe.^21^ Specifically, we clearly defined the dependent and independent variables, with the dependent variable being the presence or absence of EMS provider-perceived ADRD and the independent variables including age, sex, race/ethnicity, ZCTA-level median household income, and incident ZIP code. Additionally, our study’s design and data analysis were meticulously planned and detailed to ensure a rigorous and systematic analysis of the data.

## RESULTS

A total of 55,129 EMS patient encounters were documented among persons ≥65 years of age in San Francisco, CA, between February 1, 2020–January 31, 2022. Of these patient encounters, 51,017 (92.5%) did not indicate EMS provider-perceived ADRD. The remaining 4,112 (7.5%) did indicate EMS provider-perceived ADRD (Table 1). Among patient encounters that indicated the presence of EMS provider-perceived ADRD, the sociodemographic composition was majority female (60.4% female and 39.6% male), increased with age, except for a decline starting at 90 years, and was mostly White (42.7%) and Asian (32.3%), with smaller fractions of Black (13.5%), Hispanic (9.3%), NHOPI (1.4%), and AIAN (0.8%). Females had a higher proportion of EMS provider-perceived ADRD across all ages, compared to males, with widening sex differences starting at age 85 (Figure). The distribution of patient encounters showed a higher percentage of EMS provider-perceived ADRD in the following ZIP codes: 94112, 94109, and 94115. Of these ZIP codes, two are located in the north (94109 and 94115) and one is located in the south (94112).

**Figure.**
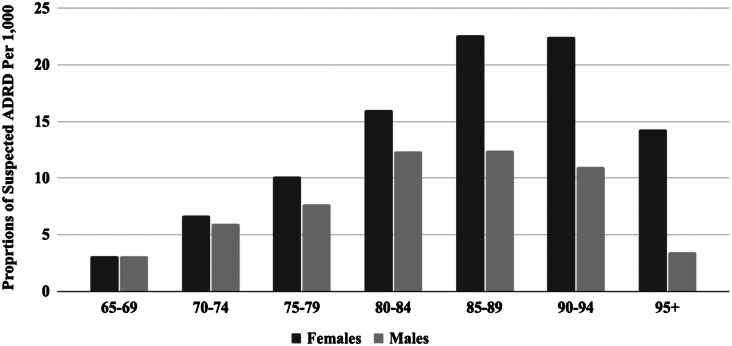
Emergency medical services provider-perceived Alzheimer’s disease and related dementias by patient age and sex in San Francisco, CA, between February 1, 2020–January 31, 2022. *ADRD*, Alzheimer’s disease and related dementias.

**Table 1.** Patient encounters^1^ for persons ≥65 years of age with and without EMS provider-perceived dementia in San Francisco, CA, between February 1, 2020–January 31, 2022.

	Absence of EMS-provider perceived ADRD (n = 51,017)		Presence of EMS-provider perceived ADRD (n = 4,112)		Total sample size (presence and absence of EMS provider-perceived ADRD) (N = 55,129)	
	Number (#)	Percentage (%)	Number (#)	Percentage (%)	Number (#)	Percentage (%)
Sex						
Female	23,599	46.3%	2,485	60.4%	26,084	47.3%
Male	27,418	53.7%	1,627	39.6%	29,045	52.7%
Age						
65–69	13,008	25.5%	171	4.2%	13,179	23.9%
70–74	10,403	20.4%	348	8.5%	10,751	19.5%
75–79	7,532	14.8%	487	11.8%	8,019	14.6%
80–84	6,894	13.5%	776	18.9%	7,670	13.9%
85–89	6,367	12.5%	951	23.1%	7,318	13.3%
90–94	4,549	8.9%	906	22.0%	5,455	9.9%
95+	2,264	4.4%	473	11.5%	2,737	5.0%
Race/Ethnicity						
White	23,694	46.4%	1,755	42.7%	25,449	46.2%
Black	9,444	18.5%	555	13.5%	9,999	18.1%
Hispanic	4,278	8.4%	383	9.3%	4,661	8.5%
Asian	12,536	24.6%	1,329	32.3%	13,865	25.2%
NHOPI^2^	621	1.2%	58	1.4%	679	1.2%
AIAN^3^	444	0.8%	32	0.8%	476	0.9%
Incident ZIP code						
94102	5,395	10.6%	208	5.1%	5,603	10.2%
94103	4,165	8.2%	103	2.5%	4,268	7.7%
94107	1,137	2.2%	67	1.6%	1,204	2.2%
94109	5,481	10.7%	368	9.0%	5,849	10.6%
94110	3,166	6.2%	209	5.1%	3,375	6.1%
94112	3,880	7.6%	525	12.8%	4,405	8.0%
94114	1,134	2.2%	58	1.4%	1,192	2.2%
94115	3,657	7.2%	350	8.5%	4,007	7.3%
94116	2,468	4.8%	302	7.3%	2,770	5.0%
94117	1,321	2.6%	154	3.8%	1,475	2.7%
94118	1,759	3.5%	201	4.9%	1,960	3.6%
94121	1,784	3.5%	203	4.9%	1,987	3.6%
94122	2,212	4.3%	236	5.7%	2,448	4.4%
94123	982	1.9%	60	1.5%	1,042	1.9%
94124	2,568	5.0%	212	5.2%	2,780	5.0%
94127	853	1.7%	67	1.6%	920	1.7%
94131	1,076	2.1%	80	2.0%	1,156	2.1%
94132	1,623	3.2%	246	6.0%	1,869	3.4%
94133	1,761	3.5%	125	3.0%	1,886	3.4%
94134	2,001	3.9%	230	5.6%	2,231	4.1%
94104, 94105, 94108, 94111, 94129, 94130, 94158^4^	2,594	5.1%	108	2.6%	2,702	4.9%

1 Patient encounters are defined as an interaction between a patient and an EMS provider. A single patient may have activated EMS multiple times during the study period. The findings represent the number of encounters, not distinct individuals.

2 NHOPI refers to Native Hawaiian or other Pacific Islander.

3 AIAN refers to American Indian or Alaska Native.

4 These ZIP codes each had less than 40 suspected ADRD cases, so they were aggregated to address problems with small sample sizes.

*ADRD*, Alzheimer’s diseases and related dementia; *EMS*, emergency medical services.

### Main Results

The first objective examines the most common primary and secondary provider impressions among patient encounters where EMS provider-perceived ADRD was recorded. A provider primary impression is defined as “the EMS personnel’s impression of the patient’s primary problem or most significant condition which led to the management given to the patient (eg, treatments, medications, or procedures).”^22^ Similarly, a secondary provider impression is defined as “the EMS personnel’s impression of the patient’s secondary problem or most significant condition which led to the management given to the patient.”^22^ Table 2 shows the top 10 most common provider primary and secondary impressions recorded for patient encounters with EMS provider- perceived ADRD. The most common primary impressions were mental disorders/altered mental status (17.1%), weakness (17.0%), and injury (15.7%), while the leading secondary impressions were general medical exam without abnormal findings (57.9%), followed by weakness (9.8%), and mental disorder/altered mental status (5.4%). However, less common outcomes (eg, gastrointestinal and cardiac) are listed in similar proportions and ranking in both the primary and secondary provider impressions.

**Table 2.** Top 10 most common EMS provider impressions for patient encounters^1^ with EMS provider-perceived Alzheimer’s disease and related dementias (N = 4,112).

EMS provider primary impression			EMS provider secondary impression		
	Number (#)	Percentage (%)		Number (#)	Percentage (%)
Mental disorder/altered mental status	703	17.1%	General medical exam without abnormal findings	2,380	57.9%
Weakness	699	17.0%	Weakness	403	9.8%
Injury	646	15.7%	Mental disorder/altered mental status	223	5.4%
Pain	537	13.1%	Injury	159	3.9%
Neurological	336	8.2%	Pain	111	2.7%
Respiratory	294	7.2%	Respiratory	96	2.3%
General medical exam without abnormal findings	268	6.5%	Neurological	90	2.2%
Gastrointestinal	104	2.5%	Gastrointestinal	72	1.8%
Cardiac	100	2.4%	Cardiac	46	1.1%
Other provider primary impression	397	9.7%	Other provider primary impression	231	5.6%
Missing	22	0.5%	Missing	301	7.3%

1 Patient encounters are defined as an interaction between a patient and an EMS provider. A single patient may have activated EMS multiple times during the study period. The findings represent the number of encounters, not distinct individuals.

*EMS*, emergency medical services.

Our second objective was to analyze EMS provider-perceived ADRD by patient sociodemographic characteristics and ZCTAs. The stepwise logistic regression models presented in Web Appendices Table 1, 2, and 3 provide detailed analyses of males and females together and separately. Table 3 shows an abridged version of the last two models from Web Appendices Table 2 and 3, stratified by sex and controlling for all covariates, including age, race/ethnicity, median household income, and incident ZIP code. Among males and females, the odds of EMS provider-perceived ADRD were higher with increasing age, except for a slight decline among males ≥95 years, a trend that was likely influenced by mortality selection. Among females, the odds of EMS provider-perceived were consistently higher for Hispanics, Blacks, Asians, and NHOPI relative to Whites. In contrast, for males, only Asians showed significantly lower odds of EMS provider-perceived ADRD relative to Whites, with no significant differences among other racial and ethnic groups.

**Table 3.** Logistic regression models^1^ for presence of EMS provider perceived ADRD among males and females by predictor specifications.^1^

	Females		Males	
	Model 1a	Model 2a	Model 1b	Model 2b
Age (ref = 65–69)				
70–74	2.52***	2.55***	2.35***	2.33***
75–79	4.39***	4.47***	4.61***	4.59***
80–84	6.59***	6.69***	8.75***	8.76***
85–89	9.26***	9.38***	10.46***	10.52***
90–94	12.36***	12.56***	13.71***	13.77***
95+	13.70***	13.97***	11.27***	11.39***
Race/ethnicity (ref = White)				
Hispanic	1.30**	1.30	1.03	.92
Black	1.20*	2.35***	1.18	1.64*
Asian	1.18**	1.41*	.87*	.78
NHOPI	1.48*	1.43	.70	.79
AIAN	1.14	1.11	.99	1.25
Median household income (ref = $147000+)				
$0–$104,399	.35***	.45**	.89	1.09
$104,400–$146,999	.34**	.43*	1.88	1.88
Race X median interaction (ref = White X $147,000)				
Black X $0–104,399		.45***		.44**
Black X $104,400–146,999		.46***		.91
Asian X $0–104,399		.81		.90
Asian X $104,000–146,999		.82		1.21
NHOPI X $0–104,399		.84		.77
NHOPI X $104,400–146,999		1.17		.89
AIAN X $0–104,399		1.11		.59
AIAN X $104,400–146,999		.98		1.00
Hispanic X $0–104,399		1.31		.84
Hispanic X $104,400–146,999		.89		1.25
Race X Median Income Interaction (*P*-value)^2^		0.01		0.02
Constant	<0.001***	<0.001***	<.001***	<.001***
Observations	26,084	26,084	29,045	29,021
AIC	15,103.15	15,101.4	11,198.68	11,195.24
BIC	15,380.90	15460.84	11,480.09	11,551.10

*** *P* < 0.001, ^**^ *P* < 0.01, ^*^ *P* < .05.

Note: Black corresponds to Black or African American.

1 All models control for incident ZIP code.

2 *P*-value for the overall joint significance of all race-by-gender interactions.

*AIAN*, *American Indian or Alaska Native;* *AIC*, Akaike information criterion; *BIC*, Bayesian information criterion; *EMS*, emergency medical services; *NHOPI,* Native Hawaiian and other Pacific Islanders.

Among females, ZCTAs with a low ($0–$104,399) and medium ($104,400–$146,999) median household income showed significantly lower odds of EMS provider-perceived ADRD, compared to ZCTAs with a high median household income (≥$147,000). In contrast, for males, no statistically significant differences were detected by median household income. Among females, the race by median household income interaction suggested that Hispanics in low- (predicted probability = .11, 95% CI 0.09–.144) and medium-income ZCTAs (predicted probability = .11, 95% CI 0.95–0.12) had a higher predicted probability of EMS provider-perceived ADRD relative to Whites in high-income ZCTAs (predicted probability = .07, 95% CI 0.06–0.08). Among males, non-statistically significant findings were found for Hispanics in low-income ZCTAs relative to Whites in high-income ZCTAs, although Hispanics in medium-income ZCTAs (predicted probability = .07, 95% CI 0.06–0.09) had a higher predicted probability of EMS provider-perceived ADRD compared to Whites in high income ZCTAs (predicted probability = .04, 95% CI 0.04–0.05). Our findings underscore the importance of patient sociodemographic characteristics (eg, sex, race, ethnicity, age, median household income), geospatial features (eg, ZCTAs), and the interplay of these factors in EMS provider-perceived ADRD in older adults in the prehospital setting.

## DISCUSSION

This study advances our understanding of the provision of prehospital care for older adults with EMS provider-perceived ADRD. Our study highlights the critical role that EMS providers’ perception and record-keeping practices may have on patients’ trajectories through other sectors of the US healthcare system. Our study suggests that the most common provider impressions recorded for older adults with EMS provider- perceived ADRD are mental disorders, weakness, and injury. These findings align with previous literature that suggests that accidents and behavioral disturbances are the most common reasons for ED visits among older adults with ADRD.^12^

Despite the insights from this study on EMS record-keeping practices, there are challenges in the identification of ADRD in the prehospital setting. It is impossible to differentiate between confirmed and perceived ADRD using documentation from the prehospital setting, and we did not have access to the electronic health records from the recipient hospitals. Furthermore, the findings from several studies suggest that the use of standardized cognitive assessments, which are commonly used in the in-hospital setting, may not be as effective in the out-of-hospital setting.^23^ ^–^ ^25^ The challenges in identifying ADRD in the prehospital setting highlight the need for enhanced EMS provider training to improve detection, emphasize the importance of developing more precise assessment tools, and underscore the need for better record-keeping practices. The efforts by San Francisco to collect ADRD-related data in the prehospital setting can also inform future efforts by other EMS agencies to improve the care of persons with ADRD along the emergency continuum.

The findings from this study suggest a higher prevalence of EMS provider-perceived ADRD in marginalized and socioeconomically disadvantaged populations, including the oldest adults, and Black, Hispanic, and Asian communities. (See Web Appendices Tables 1, 2, and 3). These results suggest that there may be health disparities in EMS provider-perceived ADRD. Future research is needed at the intersection of EMS and ADRD to ensure equitable care for older adults who access emergency care in the prehospital setting. Although this study is based on EMS provider-perceived ADRD in the prehospital setting, the findings are consistent with previous studies in the ED. For example, one study reported that older age groups and females with ADRD were more likely to use the ED.^12^ In the current study, EMS provider-perceived ADRD was also higher among older age groups and females (Table 3 and Web Appendix Table 1). Previous work suggests that Hispanic and Black older adults are 1.5 and 2 times more likely, respectively, to have ADRD than Whites.^3^ Our study supports these findings, indicating Hispanics and Black older adults have a higher likelihood of having EMS provider-perceived ADRD in their electronic patient care report, suggessting a potential higher reliance on EMS for their healthcare needs.

Future research should continue to examine the possible impacts of intersectional identities on emergency care provided in both the prehospital and in-hospital emergency settings. Delving deeper into such interactions could provide more insights for EMS training and interventions, addressing potential biases at the intersection of multiple factors such as race, ethnicity, sex, socioeconomic status, age, language, and neighborhood. Future studies should consider incorporating varied methodologies to assess whether other factors, such as knowledge of ADRD, ageism, and patient-provider language barriers, affect EMS providers’ perception of the presence of ADRD.

Leveraging EMS-ED linked data could also help compare EMS provider-perceived ADRD in the prehospital setting with clinically diagnosed ADRD in the ED. These EMS-ED data linkages are a novel approach used to study health outcomes in the emergency sector, including cardiac emergencies, opioid overdoses, and injuries.^26^ ^–^ ^28^ The ADRD research can benefit from EMS-ED data linkages by studying the provision of emergency care to older adults across the emergency continuum. Furthermore, future studies should assess the role of EMS-provided interfacility transports to older adults with ADRD who reside in long-term care facilities. The EMS-provided interfacility transports for older adults with ADRD is also an understudied area within the US healthcare system.

## LIMITATIONS

This study has several limitations. First, we examined EMS provider-perceived ADRD in the prehospital setting, which is a combination of ADRD-confirmed diagnosis disclosures by patients and caregivers, and perceived ADRD. It is impossible, however, to disentangle confirmed and perceived ADRD from the EMS documentation, and we were unable to assess the presence or absence of this health outcome in the electronic health records of the recipient hospitals. Recording the presence of ADRD, whether confirmed or perceived, is important because it may impact the course of treatment provided in the prehospital and in-hospital emergency settings and influence a patient’s trajectory through the healthcare system. Future studies should review electronic patient care report narratives to better understand the provision of emergency care for suspected and diagnosed ADRD cases.

The second study limitation is that the EMS dataset consists of patient encounters, not individual patients. A person may be represented more than once in the dataset if there were multiple 9-1-1 calls (although this limitation is present in most EMS datasets). The third study limitation is the small sample size of EMS provider-perceived ADRD across several ZIP codes (94104, 94105, 94108, 94111, 94129, 94130, 94158). To address small sample sizes, obtain reliable statistical estimates, and maintain confidentiality we aggregated the seven ZIP codes with fewer than 40 EMS provider-perceived ADRD cases. A fourth study limitation is the reliance on incident ZIP codes and ZCTAs as the geographic units of analysis; while not exactly comparable, they are the most closely aligned geographic units available. A fifth limitation is the reliance on the electronic patient care reports from the San Francisco Department of Emergency Management, which limits the generalizability of the study results to other cities or counties.

## CONCLUSION

This study advances our understanding of EMS provider-perceived Alzheimer’s disease and related dimentias in the prehospital setting, revealing sociodemographic and geographic differences among subpopulations of older adults. The findings from this study also emphasize the importance of more precise data collection in the prehospital setting, especially with a focus on ADRD.

## Supplementary Information




