# Supplementary material for: Emergency Medical Services Provider-Perceived Alzheimer’s Disease and Related Dementias in the Prehospital Setting
Source: West J Emerg Med. 2024 Nov 19;26(1):86–95. doi: 10.5811/westjem.18593 (PMC11908516; doi:10.5811/westjem.18593)
Supplement: Supplementary file 1 [file wjem-26-86-s001.docx]

**Web Appendices**

| **Table 1:  Six Stepwise Logistic Regression Models for Females and Males** | | | | | | |
| --- | --- | --- | --- | --- | --- | --- |
|  | **Model 1** | **Model 2** | **Model 3** | **Model 4** | **Model 5** | **Model 6** |
| **Age**(Ref = 65-69) | ORs | ORs | ORs | ORs | ORs | ORs |
| 70-74 | 2.54*** | 2.52*** | 2.57*** | 2.43*** | 2.44*** | 2.44*** |
| 75-79 | 4.92*** | 4.79*** | 4.94*** | 4.50*** | 4.52*** | 4.53*** |
| 80-84 | 8.56*** | 8.21*** | 8.53*** | 7.50*** | 7.54*** | 7.55*** |
| 85-89 | 11.36*** | 10.74*** | 11.24*** | 9.76*** | 9.83*** | 9.84*** |
| 90-94 | 15.15*** | 14.23*** | 14.92*** | 12.82*** | 12.93*** | 12.97*** |
| 95+ | 15.89*** | 14.55*** | 15.26*** | 13.18*** | 13.31*** | 13.34*** |
| **Gender**(Ref = Male) |  |  |  |  |  |  |
| Female |  | 1.31*** | 1.30*** | 1.28*** | 1.27*** | 1.15** |
| **Race/Ethnicity**(Ref = White) |  |  |  |  |  |  |
| Black or African American |  |  | 1.27*** | 1.19** | 1.19** | 1.15 |
| Asian |  |  | 1.08 | 1.04 | 1.05 | 0.92 |
| NHOPI |  |  | 1.24 | 1.15 | 1.15 | 0.73 |
| AIAN |  |  | 1.10 | 1.09 | 1.10 | 1.03 |
| Hispanic |  |  | 1.20* | 1.19** | 1.19* | 1.05 |
| **Incident ZIP code**(Ref = 94123) |  |  |  |  |  |  |
| 94102 |  |  |  | 1.02 | 2.31** | 2.27** |
| 94103 |  |  |  | 0.68* | 1.54 | 1.52 |
| 94107 |  |  |  | 1.16 | 1.16 | 1.14 |
| 94109 |  |  |  | 1.16 | 1.92* | 1.90* |
| 94110 |  |  |  | 1.24 | 2.05* | 2.03 |
| 94112 |  |  |  | 2.00*** | 3.30*** | 3.28*** |
| 94114 |  |  |  | 1.03 | 1.03 | 1.02 |
| 94115 |  |  |  | 1.51** | 2.48** | 2.47** |
| 94116 |  |  |  | 1.81*** | 2.98*** | 2.97*** |
| 94117 |  |  |  | 2.06*** | 2.06*** | 2.03*** |
| 94118 |  |  |  | 1.62** | 2.66*** | 2.64*** |
| 94121 |  |  |  | 1.63** | 2.68*** | 2.66*** |
| 94122 |  |  |  | 1.53** | 2.52** | 2.51** |
| 94124 |  |  |  | 1.72*** | 3.89*** | 3.88*** |
| 94127 |  |  |  | 1.15 | 1.15 | 1.15 |
| 94131 |  |  |  | 1.15 | 1.15 | 1.15 |
| 94132 |  |  |  | 2.16*** | 4.87*** | 4.85*** |
| 94133 |  |  |  | 1.19 | 2.67*** | 2.65*** |
| 94134 |  |  |  | 1.96*** | 4.42*** | 4.40*** |
| ZIP codes with less than 40 observations (90104, 90105, 94108, 94111, 94129, 94130, 94158) |  |  |  |  | 1.38 | 1.37 |
| **Median Household Income (Ref = $147000)** |  |  |  |  |  |  |
| $0 -104399 |  |  |  |  | 0.44*** | 0.45*** |
| $104400 -146999 |  |  |  |  | 0.61 | 0.61 |
| **Race X Gender**  (Ref = White X Female) |  |  |  |  |  |  |
| Black or African American X Female |  |  |  |  |  | 1.06 |
| Asian X Female |  |  |  |  |  | 1.24** |
| NHOPI X Female |  |  |  |  |  | 2.01* |
| AIAN X Female |  |  |  |  |  | 1.13 |
| Hispanic X Female |  |  |  |  |  | 1.23 |
| **Race X Gender Interaction (p-value)** |  |  |  |  |  | 0.04* |
| **Constant** | 0.01*** | 0.01*** | 0.01*** | 0.01*** | 0.01*** | 0.01*** |
| **Observations** | 55,129 | 55,129 | 55,129 | 55,129 | 55,129 | 55,129 |
| **AIC** | 26689.52 | 26627.73 | 26611.25 | 26354.27 | 26345.43 | 26343.26 |
| **BIC** | 26751.94 | 26699.06 | 26727.18 | 26648.55 | 26657.54 | 26699.96 |
| ***p<0.001, **p<0.01, *p<.05  ^1^ p-value for the overall joint significance of all race by gender interactions. | | | | | | |

| **Table 2:  Five Stepwise Logistic Regression Models for Females** | | | | | |
| --- | --- | --- | --- | --- | --- |
|  | **Model 1** | **Model 2** | **Model 3** | **Model 4** | **Model 5** |
| **Age**(Ref = 65-69) | ORs | ORs | ORs | ORs | ORs |
| 70-74 | 2.54*** | 2.62*** | 2.50*** | 2.52*** | 2.55*** |
| 75-79 | 4.52*** | 4.68*** | 4.34*** | 4.39*** | 4.47*** |
| 80-84 | 6.82*** | 7.03*** | 6.46*** | 6.59*** | 6.69*** |
| 85-89 | 9.58*** | 9.96*** | 9.08*** | 9.26*** | 9.38*** |
| 90-94 | 12.78*** | 13.35*** | 12.10*** | 12.36*** | 12.56*** |
| 95+ | 14.19*** | 14.84*** | 13.37*** | 13.70*** | 13.97*** |
| **Race/Ethnicity**(Ref = White) |  |  |  |  |  |
| Black or African American |  | 1.34*** | 1.21* | 1.20* | 2.35*** |
| Asian |  | 1.16** | 1.17** | 1.18** | 1.41* |
| NHOPI |  | 1.55** | 1.48* | 1.48* | 1.43 |
| AIAN |  | 1.15 | 1.14 | 1.14 | 1.11 |
| Hispanic |  | 1.29*** | 1.30*** | 1.30*** | 1.30 |
| **Incident ZIP code (**Ref = 94123) |  |  |  |  |  |
| 94102 |  |  | 0.80 | 2.31* | 1.85 |
| 94103 |  |  | 0.71 | 2.02* | 1.58 |
| 94107 |  |  | 0.83 | 0.83 | 0.74 |
| 94109 |  |  | 1.06 | 3.14** | 2.54* |
| 94110 |  |  | 1.10 | 3.26** | 2.77* |
| 94112 |  |  | 1.69** | 5.01*** | 4.15*** |
| 94114 |  |  | 0.83 | 0.83 | 0.78 |
| 94115 |  |  | 1.29 | 3.82*** | 3.15** |
| 94116 |  |  | 1.26 | 3.74*** | 3.05** |
| 94117 |  |  | 1.67* | 1.67* | 1.45 |
| 94118 |  |  | 1.12 | 3.30** | 2.68* |
| 94121 |  |  | 1.53 | 4.52*** | 3.66** |
| 94122 |  |  | 1.31 | 3.86*** | 3.13** |
| 94124 |  |  | 1.57* | 4.53*** | 3.91*** |
| 94127 |  |  | 1.08 | 1.08 | 1.00 |
| 94131 |  |  | 1.17 | 1.17 | 1.06 |
| 94132 |  |  | 2.10*** | 6.01*** | 4.87*** |
| 94133 |  |  | 1.04 | 2.97*** | 2.4* |
| 94134 |  |  | 1.41 | 4.02*** | 3.27*** |
| ZIP codes with less than 40 observations (90104, 90105, 94108, 94111, 94129, 94130, 94158) |  |  | 0.97 | 1.87** | 1.52 |
| **Median Household Income** (Ref = $147000) |  |  |  |  |  |
| $0-104399 |  |  |  | 0.35*** | 0.45** |
| $104400-146999 |  |  |  | 0.34** | 0.43* |
| **Race X Median Household Income Interaction**   (Ref = White X Median Household Income of $147000) |  |  |  |  |  |
| Black or African American X  $0 -104399 |  |  |  |  | 0.45*** |
| Black or African American X $104399 -146999 |  |  |  |  | 0.46*** |
| Asian X $0 -104399 |  |  |  |  | 0.81 |
| Asian X $104399 -146999 |  |  |  |  | 0.82 |
| NHOPI X $0 -104399 |  |  |  |  | 0.84 |
| NHOPI X $104399 -146999 |  |  |  |  | 1.17 |
| AIAN X $0 -104399 |  |  |  |  | 1.11 |
| AIAN X $104399 -146999 |  |  |  |  | 0.98 |
| Hispanic X $0 -104399 |  |  |  |  | 1.31 |
| Hispanic X $104399 - 146999 |  |  |  |  | 0.89 |
| **Race X Median Household Income Interaction (p-value)** |  |  |  |  | 0.01 |
| **Constant** | 0.02*** | 0.01* | 0.01*** | 0.01*** | 0.01*** |
| **Observations** | 26,084 | 26,084 | 26,084 | 26,084 | 26,084 |
| **AIC** | 15237.81 | 15219.29 | 15116.85 | 15103.15 | 15101.40 |
| **BIC** | 15294.99 | 15317.32 | 15378.26 | 15380.90 | 15460.84 |
| ***p<0.001, **p<0.01, *p<.05 | | | | | |

| **Table 3:  Five Stepwise Logistic Regression Models for Males** | | | | | |
| --- | --- | --- | --- | --- | --- |
|  | **Model 1** | **Model 2** | **Model 3** | **Model 4** | **Model 5** |
| **Age**(Ref = 65-69) | ORs | ORs | ORs | ORs | ORs |
| 70-74 | 2.48*** | 2.52*** | 2.35*** | 2.35*** | 2.33*** |
| 75-79 | 4.96*** | 5.12*** | 4.62*** | 4.61*** | 4.59*** |
| 80-84 | 9.92*** | 10.43*** | 8.76*** | 8.75*** | 8.76*** |
| 85-89 | 11.98*** | 12.67*** | 10.49*** | 10.46*** | 10.52*** |
| 90-94 | 15.73*** | 16.74*** | 13.74*** | 13.71*** | 13.77*** |
| 95+ | 12.72*** | 13.50*** | 11.32*** | 11.27*** | 11.39*** |
| **Race/Ethnicity**(Ref = White) |  |  |  |  |  |
| Black or African American |  | 1.21* | 1.18* | 1.18* | 1.64* |
| Asian |  | 0.97 | 0.87* | 0.87* | 0.78 |
| NHOPI |  | 0.82 | 0.70 | 0.70 | 0.79 |
| AIAN |  | 1.03 | 0.99 | 0.99 | 1.25 |
| Hispanic |  | 1.08 | 1.03 | 1.03 | 0.92 |
| **Incident ZIP code**(Ref = 94123) |  |  |  |  |  |
| 94102 |  |  | 1.42 | 1.60 | 1.53 |
| 94103 |  |  | 0.66 | 0.74 | 0.73 |
| 94107 |  |  | 1.77 | 1.77 | 1.72 |
| 94109 |  |  | 1.37 | 0.73 | 0.68 |
| 94110 |  |  | 1.52 | 0.81 | 0.75 |
| 94112 |  |  | 2.69*** | 1.43 | 1.32 |
| 94114 |  |  | 1.38 | 1.38 | 1.37 |
| 94115 |  |  | 1.98** | 1.05 | 0.97 |
| 94116 |  |  | 3.09*** | 1.64 | 1.54 |
| 94117 |  |  | 2.81*** | 2.81*** | 2.70*** |
| 94118 |  |  | 2.89*** | 1.53 | 1.46 |
| 94121 |  |  | 1.85* | 0.98 | 0.94 |
| 94122 |  |  | 2.04** | 1.08 | 1.02 |
| 94124 |  |  | 2.00* | 2.25 | 2.64 |
| 94127 |  |  | 1.32 | 1.32 | 1.26 |
| 94131 |  |  | 1.05 | 1.05 | 1.03 |
| 94132 |  |  | 2.25** | 2.53 | 2.27 |
| 94133 |  |  | 1.50 | 1.68 | 1.56 |
| 94134 |  |  | 3.33*** | 3.75* | 3.74* |
| ZIP codes with less than 40 observations (90104, 90105, 94108, 94111, 94129, 94130, 94158) |  |  | 0.77 | 0.67 | 0.63 |
| **Median Household Income**(Ref = $147000) |  |  |  |  |  |
| $0 -104399 |  |  |  | 0.89 | 1.09 |
| $104400 -146999 |  |  |  | 1.88 | 1.88 |
| **Race X Median Household Income Interaction**  (Ref = White X Median Household Income of $147000) |  |  |  |  |  |
| Black or African American X $0 -104399 |  |  |  |  | 0.44*** |
| Black or African American X $104399-146999 |  |  |  |  | 0.91 |
| Asian X $0 -104399 |  |  |  |  | 0.90 |
| Asian X $104399 -146999 |  |  |  |  | 1.21 |
| NHOPI X $0 -104399 |  |  |  |  | 0.77 |
| NHOPI X $104399 -146999 |  |  |  |  | 0.89 |
| AIAN X $0 -104399 |  |  |  |  | 0.59 |
| AIAN X $104399 -146999 |  |  |  |  | 1 (omitted) |
| AIAN X $147000+ |  |  |  |  | 1 (empty) |
| Hispanic X $0 -104399 |  |  |  |  | 0.84 |
| Hispanic X $104399 -146999 |  |  |  |  | 1.25 |
| **Race X Median Household Income Interaction (p-value)** |  |  |  |  | 0.02 |
| **Constant** | 0.01*** | 0.01*** | 0.01*** | 0.01*** | 0.01*** |
| **Observations** | 29,045 | 29,045 | 29,045 | 29,045 | 29,021 |
| **AIC** | 11383.24 | 11385.22 | 11198.39 | 11198.68 | 11195.24 |
| **BIC** | 11441.17 | 11484.54 | 11463.24 | 11480.09 | 11551.10 |
| ***p<0.001, **p<0.01, *p<.05 | | | | | |
